# Development of an ELISA for Evaluation of Swab Recovery Efficiencies of Bovine Serum Albumin

**DOI:** 10.1371/journal.pone.0112876

**Published:** 2014-11-17

**Authors:** Nadja Sparding, Hans-Christian Slotved, Gert M. Nicolaisen, Steen B. Giese, Jón Elmlund, Nina R. Steenhard

**Affiliations:** 1 Centre for Biosecurity and Biopreparedness, Statens Serum Institut, Copenhagen, Denmark; 2 Department of Microbiology and Infection Control, Statens Serum Institut, Copenhagen, Denmark; Kermanshah University of Medical Sciences, Iran, Republic of Islamic

## Abstract

After a potential biological incident the sampling strategy and sample analysis are crucial for the outcome of the investigation and identification. In this study, we have developed a simple sandwich ELISA based on commercial components to quantify BSA (used as a surrogate for ricin) with a detection range of 1.32–80 ng/mL. We used the ELISA to evaluate different protein swabbing procedures (swabbing techniques and after-swabbing treatments) for two swab types: a cotton gauze swab and a flocked nylon swab. The optimal swabbing procedure for each swab type was used to obtain recovery efficiencies from different surface materials. The surface recoveries using the optimal swabbing procedure ranged from 0–60% and were significantly higher from nonporous surfaces compared to porous surfaces. In conclusion, this study presents a swabbing procedure evaluation and a simple BSA ELISA based on commercial components, which are easy to perform in a laboratory with basic facilities. The data indicate that different swabbing procedures were optimal for each of the tested swab types, and the particular swab preference depends on the surface material to be swabbed.

## Introduction

After a potential biological incident with i.e. ricin, the sampling strategies and sample analysis are crucial for the outcome of the investigation and identification. In the literature there is no unambiguously preferred swab type, swabbing technique or after-swabbing treatment [1]. The recoveries obtained through swabbing depend on a number of factors such as polarity, static electricity, surface affinity, durability of the substance, sampling area, swabbing pressure applied, distribution of sample on the surface, physical and chemical properties of the surface and presence of contamination [2], [3]. The sample release from traditional swabs, such as cotton swabs, when immersed into a solution after sampling is incomplete due to trapping within the fiber matrix [1], [4]. A newer type of swab, the flocked swab, is a pre-shaped plastic applicator onto which a thin layer of nylon fiber is sprayed by a flocking process [4]. This swab has been developed to improve the recovery and release capacity because of the high surface area and the easy elution of sample due to the perpendicularly oriented fibers [5]. The instant and nearly complete (around 90%) sample release in solution through capillary action has been confirmed experimentally with the flocked swab [1], [4].

Bovine Serum Albumin (BSA) is a well-known surrogate for the toxin ricin, a potential biological weapon [6], [7]. The two proteins have similar molecular weights (BSA: 66kDA, ricin: 60 kDa A+B chains) and isoelectric points (BSA: 5.4–5.6, ricin chain B: 4.8) [6]. These factors affect chemical and physical properties such as solubility and sensitivity to denaturation of the protein [8]. Several methods have been developed to detect BSA, including counter current electrophoresis, spectrofluorimetric, label-less immunosensors, immunodiffusion, regular and sandwich ELISA [9], [10], [11], [12], [13]. For this study we needed a simple and easy detection method in order to quantify BSA in the context of dispersal and swab experiments.

The aim of this study was partly to develop a sandwich ELISA based on easily available commercial products, and partly to use the ELISA to identify optimal swab types, swabbing techniques and after-swabbing treatments for swabbing BSA. In addition, the most advantageous swabbing procedure is described, based on swabbing different surface materials.

## Materials and Methods

### ELISA setup

#### BSA standards

The standards were prepared by dissolving 32 mg BSA (Biotechnology, Grade Amresco, 0332) in 40 mL 1x-PBS solution (0.01 M phosphate buffer, 0.0027 M potassium chloride and 0.137 M sodium chloride, pH 7.4, at 25°C) (Sigma, P4417), and further diluting the solution to 80 ng/mL. In Eppendorf tubes, a twofold dilution series of 80 ng/mL to 5 ng/mL BSA were prepared. A PBS solution without BSA was used as the 0 ng/mL standard.

#### Antibodies

The antibodies tested in this study were monoclonal mouse-anti-BSA (Sigma, B2901), polyclonal rabbit-anti-BSA (Sigma, B1520), polyclonal goat-anti-serum albumin IgG (Sigma, A3812), polyclonal swine anti-rabbit immunoglobin/HRP (Dako, P0217) and goat anti-mouse immunoglobin/HRP (Dako, P0447).

#### Blocking reagents

To avoid unspecific binding, different blocking reagents were evaluated; PBST (0.1% (v/v) Tween20 (Merck, 822184) in 1x-PBS solution (Sigma, P4417)), casein diluent/blocker ready-to-use (Senova), Blocking Buffer I (AppliChem, A7099), 1% L-alanine (Sigma, A7627) in PBST and 2% unspecific anti-rabbit IgG serum (SSI, Diagnostica, A3812) in PBST.

#### Sandwich ELISA

Different reagents were tested to find the optimal setup for quantifying BSA (table 1). Maxisorp or polysorp 96-well plates (Nunc) were coated with 100 µL/well of capture antibody at 1∶400 dilution in PBS solution at 4°C overnight. The plates were washed three times with 100 µL/well PBST for 5 minutes. Blocking was performed with one of the blocking reagents and incubated at room temperature for 1 hour. For setups 10 and 14, the blocking reagent was incubated at 37°C for 1.5 hours. After an additional washing step, the BSA standards were added (100 µL/well), and the 80 ng/mL standard was added to the two controls without capture and primary antibody, respectively. Triplicates were made on the 96-well plate for each sample. Then 100 µL/well primary antibody at a 1∶400 dilution in diluting buffer (PBST) was added and incubated at room temperature for 1 hour, followed by an additional washing step. For setups 8 and 9, an additional blocking step was made with 2% L-alanine in PBST (100 µL/well), followed by a washing step. HRP conjugated primary antibody (100 µL/well) was added at a 1∶1000 dilution in diluting buffer (PBST) and incubated at room temperature for 1 hour, followed by a washing step. The bound HRP conjugate was detected by adding TMB-one ready to use (100 µL/well) (Kem-en-tec Diagnostics), and the reaction was terminated after 15 minutes by adding additional 100 µL/well 1 M H_2_SO_4_ (VWR, Fontenay-sous-Bois, France). Finally, the absorbance (A450 nm) was measured.

**Table 1:** Sandwich ELISA setups.

	Setups	CaptureAb	PrimaryAb	SecondaryAb	Blockingreagent	Different fromstandard procedure
**Polysorp**	**1**	Mouse B2901	Rabbit B1520	Swine P0217	PBST	-
	**2**	Rabbit B1520	Mouse B2901	Goat P0447	PBST	-
	**3**	Rabbit B1520	Mouse B2901	Goat P0447	L-alanine	-
**Maxisorp**	**4**	Goat A3812	Mouse B2901	Goat P0447	PBST	-
	**5**	Goat A3812	Rabbit B1520	Swine P0217	PBST	-
	**6**	Goat A3812	Rabbit B1520	Swine P0217	Anti-rabbit serum	-
	**7**	Mouse B2901	Rabbit B1520	Swine P0217	PBST	-
	**8**	Mouse B2901	Rabbit B1520	Swine P0217	PBST	Additional blocking step with anti-rabbit serum after BSA addition
	**9**	Rabbit B1520	Mouse B2901	Goat P0447	PBST	Additional blocking step with L-alanine after BSA addition
	**10**	Rabbit B1520	Mouse B2901	Goat P0447	PBST	Blocking at 37°C for 1.5 h
	**11**	Rabbit B1520	Mouse B2901	Goat P0447	Blocking Buffer I	-
	**12**	Rabbit B1520	Mouse B2901	Goat P0447	Casein	-
	**13**	Rabbit B1520	Mouse B2901	Goat P0447	Casein	Casein diluent/blocker used as dilution buffer
	**14**	Rabbit B1520	Mouse B2901	Goat P0447	L-alanine	Blocking at 37°C for 1.5 h
	**15**	**Rabbit B1520**	**Mouse B2901**	**Goat P0447**	**PBST**	**-**

Twelve different ELISA setups were tested with variations in ELISA reagents and other parameters. Antibody dilutions: capture 1∶400, primary 1∶400 and secondary 1∶1000. Ab, Antibody. The optimal ELISA setup is setup 15.

#### Optimization of capture and primary antibody concentrations

The optimal concentration of capture and primary antibody for the optimal ELISA setup (table 1, setup 15) was determined with a fixed concentration of secondary antibody (1∶1000). Dilutions of capture antibody at 1∶400 and 1∶800 were tested in combination with primary antibody at 1∶400, 1∶800 and 1∶1600. In addition, a combination of capture and primary antibody at 1∶1600 was tested.

#### Commercial BSA ELISA kit

A BSA ELISA kit (Alpha Diagnostic Intl., San Antonio, USA, 80100) was used as a reference to the developed BSA sandwich ELISA and to verify the BSA content of the BSA standards used for the ELISA developed in this study. Instructions were followed as described in the kit manual. Kit BSA standards (80, 40, 20, 5, 1 and 0 ng/mL) and BSA standards (two-fold serial dilutions of 40–0.625 and 0 ng/mL) were analyzed on the ELISA in duplicate.

### Swabbing test

#### Swabbing technique

The optimal swabbing procedure (swabbing technique and after-swabbing treatment) was tested on a nonporous surface material (plastic). A volume of 100 µl of a 100 mg/L BSA (Biotechnology, Grade Amresco) water solution was applied to 36 plastic test squares (10×10 cm) in 5 drops of 20 µl and distributed evenly on the surface areas using a drigalski spatula. Water was applied in the same way to 12 additional plastic test squares and used as negative controls (one for each combination of swab type, swabbing technique and after-swabbing treatment). The applied BSA solution was left to dry overnight. Two types of swabs were tested; a cotton gauze swab (Cura Care, 10×10 cm, 8 layers, sterile) handled with a pair of sterile peans and a regular flocked nylon swab (Copan Diagnostic, 502CS01). Swabbing was performed by the same person during the experiment to ensure equal swabbing pressure. The swabs were swept in horizontal and vertical sweeps, turning the swab when changing direction, to cover the test squares once. After swabbing, the gauze swab was transferred to a 50 mL centrifuge tube and the flocked swab to an Eppendorf tube, containing 3 mL and 1 mL PBS, respectively. For both swab types three swabbing techniques were applied (I–III); swabbing with a single PBS pre-moistened swab (I), PBS pre-moistened swab followed by a dry swab (II) or rinse of the area by pipetting with 1 mL water added in droplets followed by a dry swab (III).

#### Swab treatment and recovery

After swabbing, three after-swabbing treatments were applied to the gauze swab (A–C); squeezing the gauze swab by hand (A), squeezing the gauze swab in a syringe (B) or by adding 2 mL PBS followed by massaging the gauze swab with a pair of tweezers (C). For the flocked swab only vortexing of the Eppendorf tube (D) was applied. The swabs were removed immediately after the swab treatment. Quantification of the BSA content in each sample was done in triplicate by the sandwich ELISA (table 1, setup 15).

#### Surface recoveries

The combination of swabbing techniques and after-swabbing treatments that showed the highest recovery of BSA from plastic were selected to further test the recovery from other surface materials. Combination I and B for the gauze swab and combination II and D for the flocked swab were used to determine the swab recoveries from envelopes, painted metal, laminate, glass, untreated wood and concrete, in addition to plastic. Again, one negative water control was made for each swab type on all surface materials. The recoveries were quantified in triplicate by the sandwich ELISA.

#### Data analysis and statistics

Results are expressed as the mean ± standard deviation (SD) of *n* separate experiments. The two data groups obtained by the developed and commercial ELISA were compared in GraphPad Prism 5 using a t-test comparing the slopes of the regression lines.

The swab recovery efficiencies were calculated by dividing the amount of BSA from swab sample with the known dispersed amount of BSA. The significance of difference between groups was evaluated by one-way analysis of variance (ANOVA) followed by a Dunnett’s test or with two-tailored, unpaired t-test in GraphPad Prism 5.

## Results

### ELISA setup

#### Sandwich ELISA development and optimization

**Figure 1:**
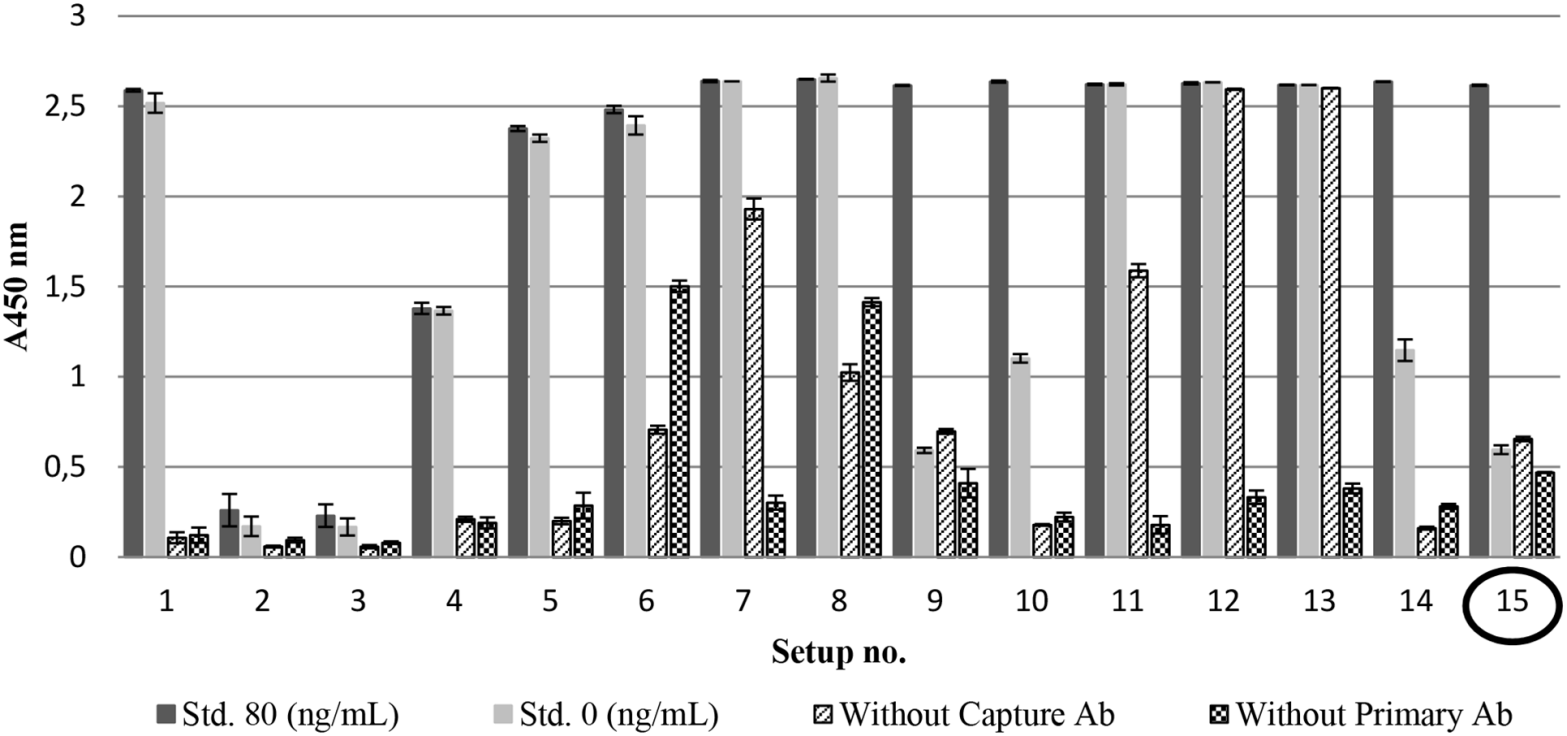
Sandwich ELISA setups. Different ELISA setups (table 1, setups 1–15) were tested based on absorbance measured at 450 nm for the endpoint BSA standards (std. 80 and 0 ng/mL), and the controls without capture antibody (Ab) and primary antibody (Ab). The parameters for each setup are listed in table 1. Data is expressed as mean ± SD (n = 3).

Different ELISA setups (table 1, setup 1–15) were tested and evaluated based on the data presented in figure 1. Two setups (table 1, setups 9 and 15) showed a maximum A450 nm value for the 80 ng/mL BSA standard and an A450 nm value <1 for the 0 ng/mL BSA standard. Since there was no obvious difference between the results from these two ELISA setups, and the only difference between them in the protocol was an additional blocking step with L-alanine after BSA addition for setup 9, the more simple protocol for setup 15 was preferred.

#### Different combinations of capture and primary antibody dilutions for ELISA setup 15

**Figure 2:**
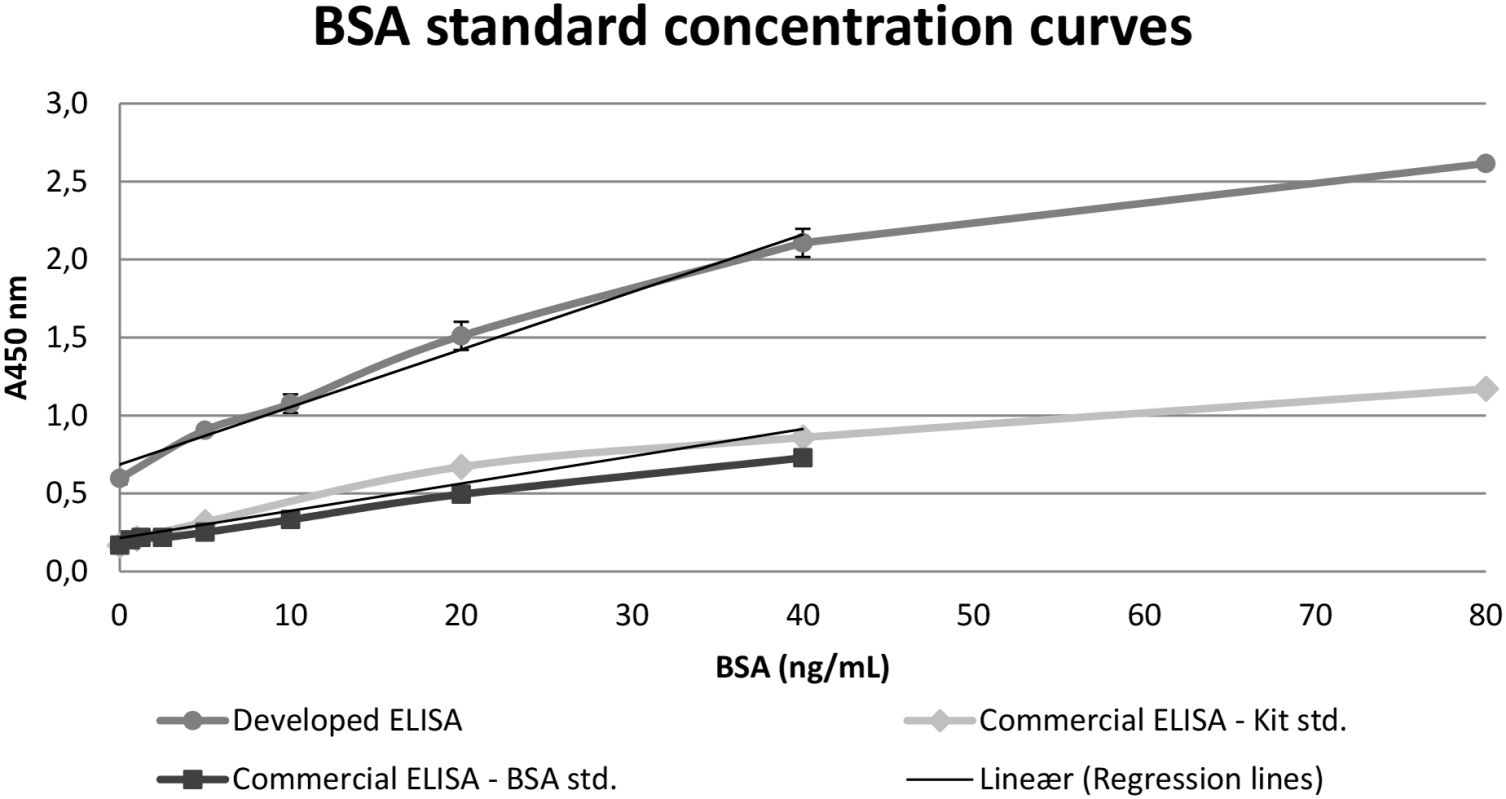
Concentration curves for developed and commercial BSA ELISAs. The optimal sandwich ELISA (table 1, setup 15) including the in-house BSA standards (std.) from 0–80 ng/mL (Developed ELISA). Capture antibody at a 1∶400 dilution, primary antibody at a 1∶800 dilution and secondary antibody at a 1∶1000 dilution. With the commercial BSA ELISA the in-house BSA standards (Commercial ELISA – BSA std.) were tested in addition to the commercial kit BSA standards (Commercial ELISA – Kit std.). There were no statistically significant difference (t-test comparing the slopes of the regression lines, p≥0.05) between the datasets of the BSA concentration curve of the developed (y = 0.0368 · x+0.686, r = 0.9853) and commercial ELISA (y = 0.0175·x+0.2144, r = 0.9534) in BSA concentration ranging from 0–40 ng/mL. Data is expressed as mean ± SD (n = 3) or mean (n = 2).

The measuring range for the dilution combinations of capture and primary antibody (dilution of capture antibody/dilution of primary antibody) is the maximum absorbance minus the background absorbance at A450 nm and were 2.02 for (1∶400/1∶400), 2.08 for (1∶400/1∶800), 1.08 for (1∶400/1∶1600), 1.63 for (1∶800/1∶400), 1.92 for (1∶800/1∶800), 1.57 for (1∶800/1∶1600) and 1.35 for (1∶1600/1∶1600). Dilution (1∶400/1∶800) was found to be the optimal combination of capture and primary antibody for quantification within the BSA standard concentration range (0–80 ng/mL), and was selected for quantification in this study. Under these conditions, the standard concentration curve was a linear function at a concentration range from 0–40 ng/mL BSA (figure 2). The detection limit was defined as the background absorbance (A450 nm) plus 2 SD of the linear equation. Therefore, the developed ELISA can be considered to have a sensitivity of 1.32 ng/mL for BSA.

The BSA content of the standards was quantified with the commercial reference ELISA kit and showed an equal content of BSA in the standards made in this study and the standards from the commercial kit. In addition there is no statistically significant difference (p≥0.05) between the datasets of the BSA concentration curve of the developed and commercial ELISA (figure 2).

### Swabbing test

#### Optimal sampling procedure

**Figure 3:**
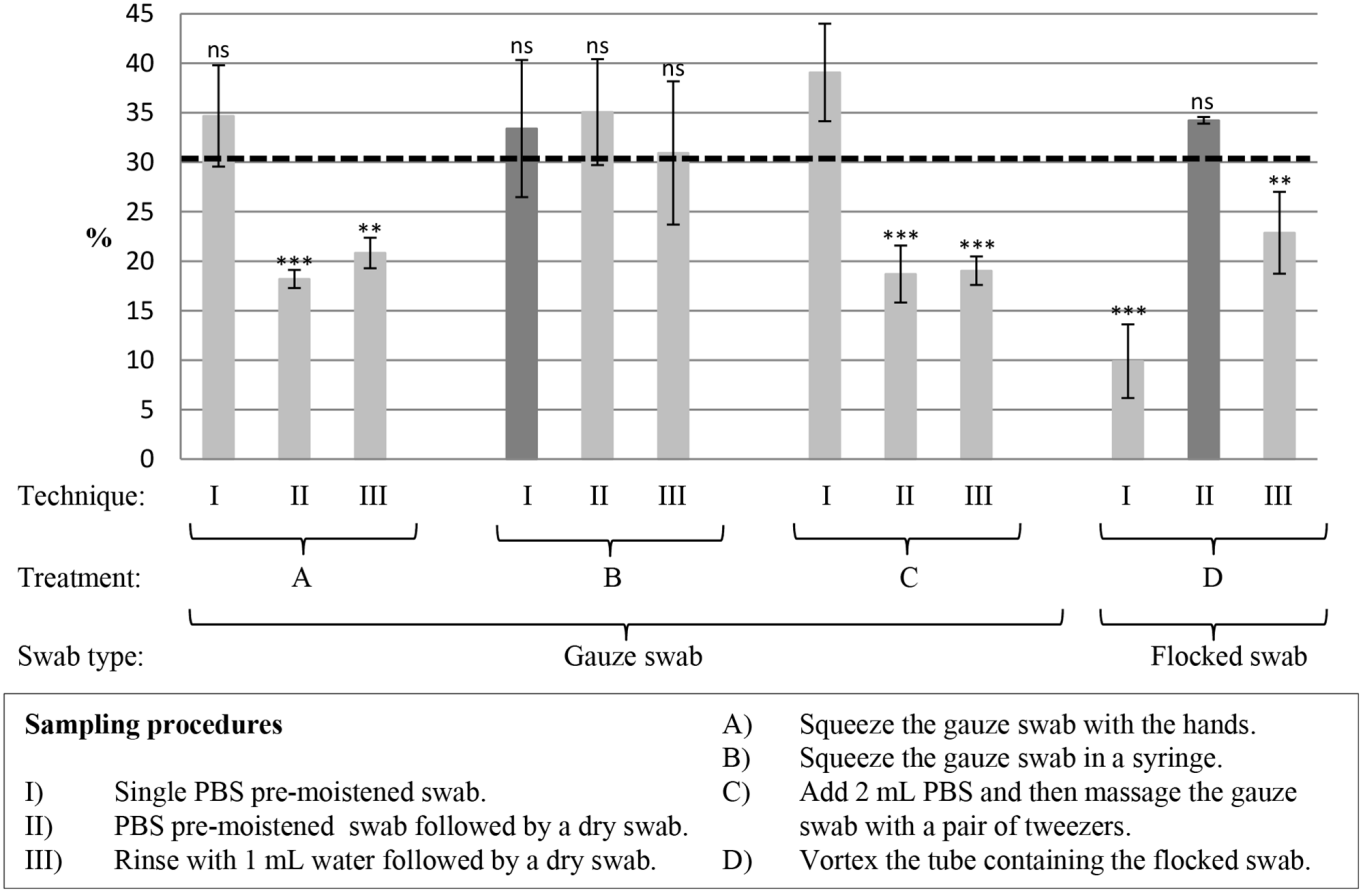
Recoveries from different combinations of swabbing techniques and after-swabbing treatments. The different combinations of swabbing techniques and swab treatments were tested on a plastic surface material with gauze (in 3 mL PBS) and flocked (in 1 mL PBS) swabs. Six setups have a mean recovery >30% and they are not significantly different from the highest mean recovery (gauze cotton swab combination I/C). Data is expressed as mean ± SD (n = 3), one-way ANOVA, Dunnett’s test for post-hoc comparison vs. gauze I/C (the highest mean recovery), ns (≥0.05) not significant, ** significant at p<0.005 and *** significant at p<0.001.

Tested on plastic test squares, we compared the recoveries of BSA obtained by different combinations of swabbing techniques and after-swabbing treatments for the two tested swab types. Combinations resulting in a mean recovery of minimum 30% were selected. For the gauze swab, combinations I A–C, II B, and III B were above 30%. The recoveries for these combinations are not significantly different (p≥0.05) for the combination with the highest recovery, gauze swab I/C (figure 3). As swabbing technique I and after-swabbing treatment B are the simplest procedures to perform, this combination was preferred for the gauze swab. In addition, it was possible to extract the largest sample volume from the swab with treatment B (data not shown). Only one swabbing technique, II, was included for the flocked swabs under the selected criteria combined with the treatment for flocked swabs (D).

#### Surface recoveries

**Figure 4:**
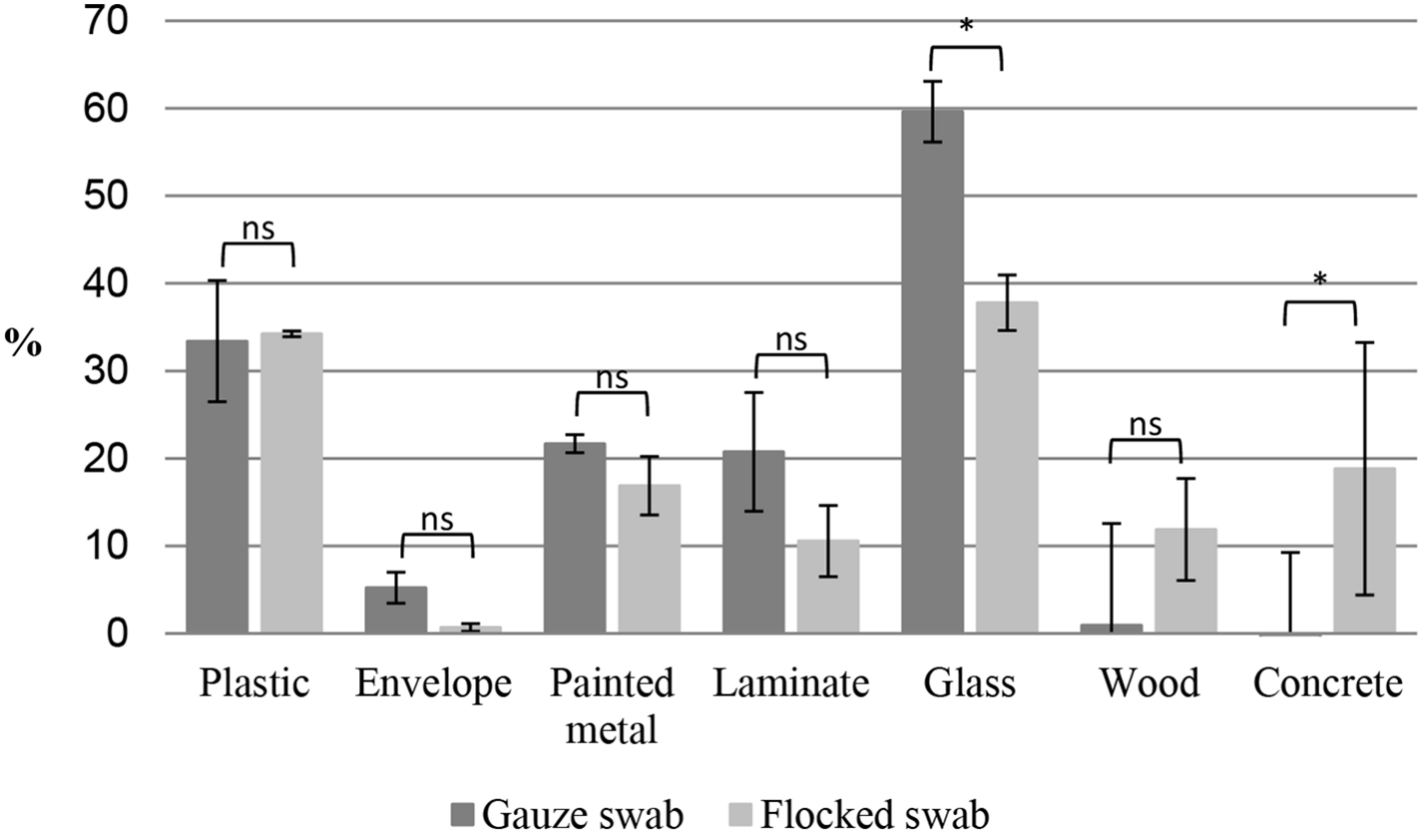
Recovery efficiencies of BSA from seven different surfaces. The figure shows the recovery from different surface materials expressed as a percentage of the known amount of BSA. The surfaces are swabbed with gauze cotton swabs (I/B, figure 3) and flocked nylon swabs (II/D, figure 3) respectively. Data is expressed as mean ± SD (n = 3), two-tailored, unpaired t-test, ns (≥0.05) not significant and * significant at p<0.05.

Recoveries from the different surface materials varied from around 0–60%. The highest yields were obtained from nonporous surfaces as plastic and glass. Whereas lower yields were seen from the porous materials (figure 4). Comparing the mean recoveries obtained with the gauze cotton swab and the flocked nylon swab there were no significant difference (p≥0.05) in the performance of either of the swab types when looking at the seven surface materials individually, with expectation of glass and concrete. The gauze swabs seem to yield a higher recovery than the flocked swabs from glass surfaces (nonporous material) whereas flocked swabs performed better on concrete (porous material) (figure 4). When comparing the swab types individually there was a statistical significant difference between the recoveries obtained from many of the surfaces compared to the plastic surface material (table 2).

**Table 2:** Comparison of the swab recoveries from different surface materials.

Surfacematerial	Description	Significance withgauze swab	Significance withflocked swab
**Plastic**	Nonporous	-	-
**Envelope**	Porous	*	***
**Painted metal**	Nonporous	ns	*
**Laminate**	Porous	ns	**
**Glass**	Nonporous	ns	ns
**Wood**	Porous	*	**
**Concrete**	Porous	**	ns

Each surface material is listed with a visual description of the material and statistical significance compared to the plastic surface material. The comparison is based on data present in figure 4, one-way ANOVA, Dunnett’s test for post-hoc comparison vs. plastic (material used to evaluate the swab procedures), ns (p≥0.05) not significant, *significant at p<0.05, **significant at p<0.005 and ***significant at p<0.001.

## Discussion

This study presents a BSA sandwich ELISA with a quantification range from 1.32–40 ng/mL for quantification of BSA from swab samples (figure 2). The ELISA has comparably sensitivity to other BSA sandwich ELISAs, both commercial assays and assays described in the literature [10], [11], [12]. The benefits of using the described ELISA are that the setup is simple and all components are commercially available. Regular BSA ELISAs have been developed, but the sandwich ELISAs seem to be more advantageous for the detection of BSA in samples containing other proteins [11]. The optimal ELISA setup is the assay that presents the highest difference between the upper concentration limit of the BSA standards (80 ng/mL) and the assay background (0 ng/mL). The background absorbance of our ELISA is slightly higher than that of other assays; however, the level is acceptable as the maximum absorbance is at an equally higher level. We tested different blocking dilutions in addition to PBST, such as rabbit serum and blocking at 90 min. at 37°C as described by Zhang et al. [12]. Although different attempts to decrease non-specific binding failed, PBST blocking was found to be the optimal blocking agent tested in this study. In some of the ELISA setups (9 and 15) in figure 1, the absorbance of the control without primary antibody (with BSA) is lower than the 0 ng/mL BSA standard. This could indicate that the BSA has a blocking effect in these assay setups. BSA is a widely used as a blocking reagent in other ELISAs [14], [15].

The BSA sandwich ELISA (table 1, setup 15) developed in this study was used to quantify BSA swab recoveries from different surface materials and to evaluate the most optimal swab type, swabbing technique and after-swabbing treatment. Different combinations of swab type, technique and treatment yielded the highest recovery in this study. The optimal combinations are I/B and II/D for the gauze and flocked swab, respectively (figure 3). Since the most attributable sampling protocol for the gauze and flocked swab were determined for plastic surface material, each method (combination of swabbing technique and after-swabbing treatment) should be evaluated together with each surface material. The issue of which swab type to use depends on the material of the surface to be swabbed as well as the analytic method used to analyze the swab samples. In the literature, the general understanding is that the presence of cotton fibers or impurities associated with the cotton swabs might inhibit PCR [3] and hence gauze swabs can result in analytic problems. The gauze swabs used in this study showed no hindering of (unpublished data). The synthetic nylon material of the flocked swabs is expected to have no interference with analytic methods.

In this study we applied a BSA solution to the surface test squares and not BSA as a dry formulate. For scenarios where BSA, a surrogate for ricin, is dispersed as a dry powder, the protein may be more easily removed from a surface than in solution [16]. Hence the BSA recoveries obtained in this work, ranging from 0–60%, may be higher for weapon-grade powder. The highest recovery was observed from glass and plastic with both gauze and flocked swabs, and overall the recoveries decrease with the absorbency and porosity of the material of the surfaces, with the lowest recovery from envelope (figure 4 and table 2). Overall the gauze and flocked swabs seem to perform equally well, gauze swabs may perform better than flocked swabs on nonporous surfaces and vice versa for porous surfaces. The relatively high standard deviations, observed in many cases (figure 4), from swabbing the different surface materials in this study are consistent with previous work by Rose et al. [3].

The flocked swab is physically smaller than the gauze swab and still gain recoveries in the same range. It might be that the gauze swab collects more sample material than the flocked swab but that the flocked swab has a higher release capacity. The two swab types seem equally easy to use; the flocked swabs are easier to use for small areas and gauze swabs are easier to use for bigger areas. The price is the only factor that gives an unequivocal answer to which swab to prefer since the flocked swab is more expensive.

Frawley et al. [2] studied the recovery efficiencies of ricin, in addition to anthrax spores, recovered from polyester swabs and gauze wipes. From wet polyester swab they obtained 2.5% (plastic) and 2.1% (untreated wood) ricin and 2.5% (plastic) and 1.4% (untreated wood) ricin from the wet gauze wipe. In comparison we obtained 34.2% and 11.9% BSA from the flocked nylon swab and 33.4% and 0.9% BSA with the gauze cotton swab. Our recovery efficiencies are generally higher than the efficiencies presented in the study by Frawley et al. [2] but when looking at their anthrax spore recoveries they are overall lower than other anthrax spore recovery efficiencies found in other studies [3], [16], [17], [18]. All in all, there is a great deal of literature available concerning sampling of spores, while the amount of data on ricin seems sparser which makes comparison between studies difficult. The difference in the properties of ricin and BSA may contribute to the variations in swab recoveries of these two substances. Furthermore, when comparing different swab studies one should consider that the same type of surface material may vary as may other factors as variation in sampling area, applied swabbing pressure, distribution of sample on the surface, physical and chemical properties of the surface, and presence of contamination [3]. The presence of contamination may influence the recovery efficiencies of BSA as previously seen for ricin, where swabbing of spores were found to be less affected by contamination [2].

In conclusion, this study presents a swabbing procedure evaluation and a simple BSA ELISA based on commercial components. The ELISA showed similar sensitivity as the tested commercial reference BSA ELISA kit. Furthermore, the study showed recovery efficiencies for swabbing procedures and from a series of surface materials. In the presented data there was no unambiguous standard swabbing procedure to prefer and it is suggested that a decision should be made on the spot depending on the surface one needs to swab. If possible smooth, nonporous surfaces should be selected for swabbing. Based on the results in this study it is recommended to incorporate both swab types in the swabbing procedure so that the choice of which one to be used can be made on the spot depending on the available surfaces. The gauze swab should be used for nonporous surfaces and the flocked swab for porous surfaces.
